# A Natural Language Processing Model for COVID-19 Detection Based on Dutch General Practice Electronic Health Records by Using Bidirectional Encoder Representations From Transformers: Development and Validation Study

**DOI:** 10.2196/49944

**Published:** 2023-10-04

**Authors:** Maarten Homburg, Eline Meijer, Matthijs Berends, Thijmen Kupers, Tim Olde Hartman, Jean Muris, Evelien de Schepper, Premysl Velek, Jeroen Kuiper, Marjolein Berger, Lilian Peters

**Affiliations:** 1 Department of Primary- and Long-Term Care University Medical Center Groningen Groningen Netherlands; 2 Data Science Center in Health University Medical Center Groningen Groningen Netherlands; 3 Department of Medical Microbiology and Infection Prevention University Medical Center Groningen Groningen Netherlands; 4 Department of Medical Epidemiology Certe Foundation Groningen Netherlands; 5 Department of Primary and Community Care Radboud University Nijmegen Medical Center Nijmegen Netherlands; 6 Care and Public Health Research Institute Department of Family Medicine Maastricht University Medical Center Maastricht Netherlands; 7 Department of General Practice Erasmus Medical Center Rotterdam Netherlands; 8 Municipal Health Service Groningen Groningen Netherlands; 9 Midwifery Science, Amsterdam Public Health Vrije Universiteit Amsterdam Amsterdam University Medical Center Amsterdam Netherlands

**Keywords:** natural language processing, primary care, COVID-19, EHR, electronic health records, public health, multidisciplinary, NLP, disease identification, BERT model, model development, prediction

## Abstract

**Background:**

Natural language processing (NLP) models such as bidirectional encoder representations from transformers (BERT) hold promise in revolutionizing disease identification from electronic health records (EHRs) by potentially enhancing efficiency and accuracy. However, their practical application in practice settings demands a comprehensive and multidisciplinary approach to development and validation. The COVID-19 pandemic highlighted challenges in disease identification due to limited testing availability and challenges in handling unstructured data. In the Netherlands, where general practitioners (GPs) serve as the first point of contact for health care, EHRs generated by these primary care providers contain a wealth of potentially valuable information. Nonetheless, the unstructured nature of free-text entries in EHRs poses challenges in identifying trends, detecting disease outbreaks, or accurately pinpointing COVID-19 cases.

**Objective:**

This study aims to develop and validate a BERT model for detecting COVID-19 consultations in general practice EHRs in the Netherlands.

**Methods:**

The BERT model was initially pretrained on Dutch language data and fine-tuned using a comprehensive EHR data set comprising confirmed COVID-19 GP consultations and non–COVID-19–related consultations. The data set was partitioned into a training and development set, and the model’s performance was evaluated on an independent test set that served as the primary measure of its effectiveness in COVID-19 detection. To validate the final model, its performance was assessed through 3 approaches. First, external validation was applied on an EHR data set from a different geographic region in the Netherlands. Second, validation was conducted using results of polymerase chain reaction (PCR) test data obtained from municipal health services. Lastly, correlation between predicted outcomes and COVID-19–related hospitalizations in the Netherlands was assessed, encompassing the period around the outbreak of the pandemic in the Netherlands, that is, the period before widespread testing.

**Results:**

The model development used 300,359 GP consultations. We developed a highly accurate model for COVID-19 consultations (accuracy 0.97, *F*_1_-score 0.90, precision 0.85, recall 0.85, specificity 0.99). External validations showed comparable high performance. Validation on PCR test data showed high recall but low precision and specificity. Validation using hospital data showed significant correlation between COVID-19 predictions of the model and COVID-19–related hospitalizations (*F*_1_-score 96.8; *P*<.001; *R*^2^=0.69). Most importantly, the model was able to predict COVID-19 cases weeks before the first confirmed case in the Netherlands.

**Conclusions:**

The developed BERT model was able to accurately identify COVID-19 cases among GP consultations even preceding confirmed cases. The validated efficacy of our BERT model highlights the potential of NLP models to identify disease outbreaks early, exemplifying the power of multidisciplinary efforts in harnessing technology for disease identification. Moreover, the implications of this study extend beyond COVID-19 and offer a blueprint for the early recognition of various illnesses, revealing that such models could revolutionize disease surveillance.

## Introduction

Health care has been transformed by artificial intelligence (AI), leading to remarkable breakthroughs in diagnosis and treatment [1-3]. Among the most commonly used methods is natural language processing (NLP), a form of AI that focuses on understanding and interpreting human language. NLP can extract meaningful information from unstructured data such as free-text fields by recognizing patterns and relationships between words in a sentence [4]. One such NLP technique is the bidirectional encoder representations from transformers (BERT) model, which can be fine-tuned with an additional output layer for a specific task [5]. For instance, a BERT model can be adjusted for disease identification in electronic health records (EHRs), allowing it to analyze and interpret large amounts of data to identify patterns indicative of a particular disease.

General practitioners (GPs) serve as gatekeepers to specialist care in the Dutch health care system, making them the first point of contact for most patients and giving them a key role in recording patient health information in EHRs. This includes linking an International Classification of Primary Care (ICPC) code to each entry [6]. However, the absence of a specific ICPC code for COVID-19 and the limited availability of suitable testing at the outbreak of the COVID-19 pandemic highlighted the difficulty in identifying relevant patient consultations in GP EHRs [7-9]. Extracting meaningful information from medical free-text fields has also proven difficult [4]. Nevertheless, accurate health surveillance requires the identification of COVID-19 consultations not only during the acute phase of the pandemic but also in retrospect (eg, to evaluate post-COVID trajectories). The use of NLP in health care has the potential to revolutionize disease identification, but significant challenges need to be overcome before this potential can be realized. A key challenge is ensuring that NLP models are robust, reliable, and safe for use in medical practice. This requires a rigorous approach to model development and validation.

For this study, we found BERT to employ the most advanced NLP technique available to analyze GP registration texts and predict COVID-19 cases. BERT’s key superiority lies in its ability to capture contextual information from both preceding and succeeding words, enabling a deeper understanding of complex medical language and context. To the best of our knowledge, this has not been done on Dutch GP registration texts until now. Additionally, and more technically, BERT’s pretrained architecture on extensive corpora ensures superior feature extraction and generalization, empowering our model to detect subtle nuances in GP texts and predict COVID-19 cases with potentially very high precision. BERT models in medical literature have been shown to improve performance in common clinical NLP tasks such as named entity recognition and medical natural language inference [10]. By leveraging the capabilities of BERT over traditional NLP methods, our study aims to significantly advance the field of medical informatics and contribute to more effective disease surveillance and management strategies.

We aimed to develop a BERT model that could accurately identify COVID-19 GP consultations from unstructured text in the EHRs of Dutch general practice, thereby validating the generalizability and applicability of this approach in different settings. This innovative approach addresses the urgent need to identify COVID-19 consultations accurately from unstructured text in general practice EHRs. We anticipate that the comprehensive methodology described in this study will lay the groundwork for the development of reliable and robust NLP models, which in turn, could revolutionize disease identification in general practice and improve patient outcomes.

## Methods

### Data Source and Study Population

The required data were extracted from the EHRs of general practices participating in research networks managed by 3 university medical centers in the Netherlands. These databases contain routinely collected data from longitudinal EHRs. The networks were the Academic GP Development Network (Academisch Huisartsen Ontwikkel Netwerk [AHON]) managed by the University Medical Center Groningen, which includes 59 practices in the north of the Netherlands; the Family Medicine Network managed by Radboud University Medical Center Nijmegen, which includes 6 practices in the east; and the Research Network Family Medicine managed by Maastricht University Medical Center, which includes 28 practices in the south. The EHRs covered a period from January 1, 2019, to December 31, 2021. A GP consultation was defined as any consultation between a patient and either a GP or a practice nurse, which had at least one ICPC code and a corresponding free-text note recorded in the EHR. All Dutch GPs use the country-specific ICPC-1 managed by the Dutch College of GPs [11]. To define the study database, we manually assessed existing ICPC-1 codes to develop a list of all codes that could be related to acute COVID-19 infection. This list was created by a GP (MH) and a microbiologist (M Berends), and all GP consultations linked to at least one of these codes were extracted (Multimedia Appendix 1).

### Ethical Considerations

Data from the registry databases were pseudonymized such that the researchers could not access individual patient details. The medical research ethics committee of the University Medical Center Groningen determined that this research did not require ethics approval, according to Dutch law (Medical Research Involving Human Subjects Act 2020/309).

### BERT Model Development and Testing

**Figure 1:**
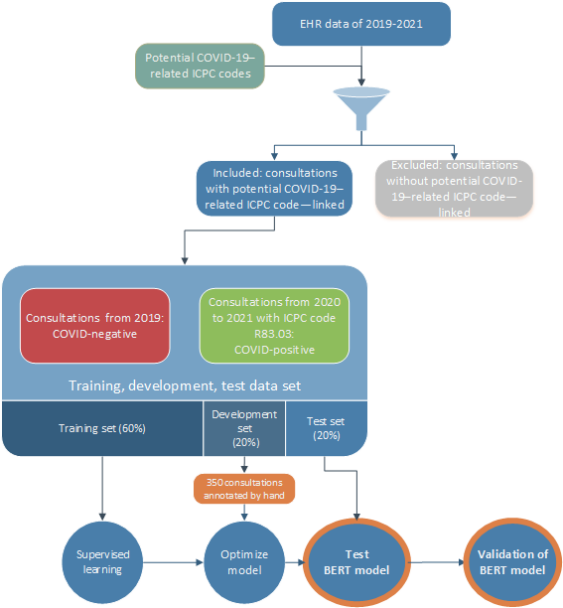
Flowchart for the composition of the database and composition of the bidirectional encoder representations from transformers model. BERT: bidirectional encoder representations from transformers; EHR: electronic health record; ICPC: International Classification of Primary Care.

A BERT model was developed to classify GP consultations as COVID-19 or non–COVID-19 by using an open source, pretrained, Dutch BERT model [12]. This neural network model could effectively interpret the contextual relationships between words in a sentence if written in Dutch [12]. The pretrained model was fine-tuned for the classification task by adding neural network layers before training the whole model to perform the specific task of identifying COVID-19 GP consultations. This approach leveraged the language understanding capabilities of the base model, while tailoring it to the specific task of classifying GP consultations. Details regarding the development and performance of our model can be found in Multimedia Appendix 2. A stepwise approach was followed to fine-tune the BERT model. We divided the data set into 3 parts, using 60% for the training set, 20% for the development set, and 20% for the test set (Figure 1).

1. Training set: Supervised learning uses a labeled data set to train the model. We labeled all GP consultations included from 2019 but before the outbreak of the pandemic as conclusively negative COVID-19 GP consultations (label 0) and consultations labeled with ICPC code R83.03 (confirmed SARS-CoV-2) as conclusively positive COVID-19 GP consultations (label 1). The COVID-19–specific ICPC code R83.03, introduced in the Netherlands in November 2020, came with the instruction that all GPs should use the code for confirmed COVID-19 consultations [7]. During the training step, the BERT model did not have access to corresponding ICPC codes to ensure that it only had access to the EHR text and could not use the ICPC codes to differentiate between COVID-19–positive and COVID-19–negative consultations.
2. Development set: Model performance was evaluated using the development set. Two independent researchers (MH and M Berends) labeled 350 GP consultations as related or not related to COVID-19 before comparing their results with the same consultations used in the BERT model. The final model was chosen based on a sigmoid plot and relevant metrics (ie, accuracy, F_1_-score, precision, recall, and specificity).
3. Test set: To assess the accuracy of the final BERT model, its performance was evaluated on new data (the test set).

### Evaluation of Generalizability and Applicability

Following the development and testing of the fine-tuned BERT model, its performance was validated with 3 different methods that assessed its generalizability in practical applications. Each validation method offered different insights into the model’s ability to classify COVID-19–related GP consultations accurately and consistently across data sets.

#### External Validation

The generalizability of the final BERT model to a different geographic region with a similar health care system was assessed through an EHR data set with the same structure as the training data. We relied on data from the Rijnmond Primary Care database, managed by the Department of General Practice of the Erasmus Medical Center, University Medical Centre Rotterdam, which covered the west of the Netherlands. The Rijnmond Primary Care database included GP consultations from 2019 with potential COVID-19–related ICPC codes (negative COVID status) and GP consultations with ICPC code R83.03 (positive COVID status).

#### Validation Using Polymerase Chain Reaction Tests

In this validation step, the performance of the BERT model was assessed using GP consultations from before the introduction of the specific ICPC code for COVID-19. We used data from the municipal health services and linked these to the AHON database to include patients who underwent polymerase chain reaction (PCR) testing for COVID-19 around the time of a GP consultation with a potential COVID-19–related ICPC code (7 days before to 7 days after the date of testing). GP consultations with a positive PCR test result during this 2-week window were labeled as related to COVID-19, while GP consultations with a negative PCR test were labeled as not related to COVID-19. For patients who underwent multiple testing and had both positive and negative PCR test results within the defined 2-week window, we considered them COVID-positive and having symptoms related to COVID-19.

#### Validation Before the Introduction of a COVID-19–Specific ICPC Code

To evaluate the model’s ability to identify COVID-19 consultations in the data from before the introduction of a COVID-19–specific ICPC code, its predictions were compared with data for COVID-19 hospital admissions in the Netherlands during the first year of the pandemic based on open data from the National Institute for Public Health and the Environment [13]. This step improved our understanding of the model’s utility in medical settings.

### Statistical Analysis

This study’s primary outcome was the performance of the BERT model in identifying health care consultations related to COVID-19 in the test set, evaluated using performance metrics and sigmoid plots. External validation and PCR test validation were assessed using descriptive statistics, performance metrics, and sigmoid plots, while the comparison with COVID-19–related hospital admissions in 2020 was done by linear regression modeling. For the linear regression, we set the dependent variable as the weekly number of hospital admissions for COVID-19 in 2020 and the independent variable as the predicted COVID-19 consultations generated by the BERT model, using a significance level of *P*<.05. Sigmoid plots and performance metrics were analyzed using Python software, and additional analyses were conducted using R software (version 4.1.1; R Foundation for Statistical Computing) [14].

## Results

### Data Source and Study Population

This study included 300,359 GP consultations extracted based on ICPC codes potentially related to COVID-19 or ICPC code R83.03 for confirmed SARS-CoV-2 (COVID-19). Among these, 251,362 consultations (83.7%) from 2019 had at least one ICPC code potentially related to COVID-19, while 48,997 (16.3%) had the ICPC code R83.03. The AHON database provided 191,508 consultations (63.8%) of 184,700 patients, the Family Medicine Network database provided 29,409 consultations (9.8%) of 31,351 patients, and the Research Network Family Medicine database provided 79,442 consultations (26.4%) of 87,499 patients. Additional population characteristics can be found in Multimedia Appendix 3.

### Testing the Developed BERT Model

**Figure 2:**
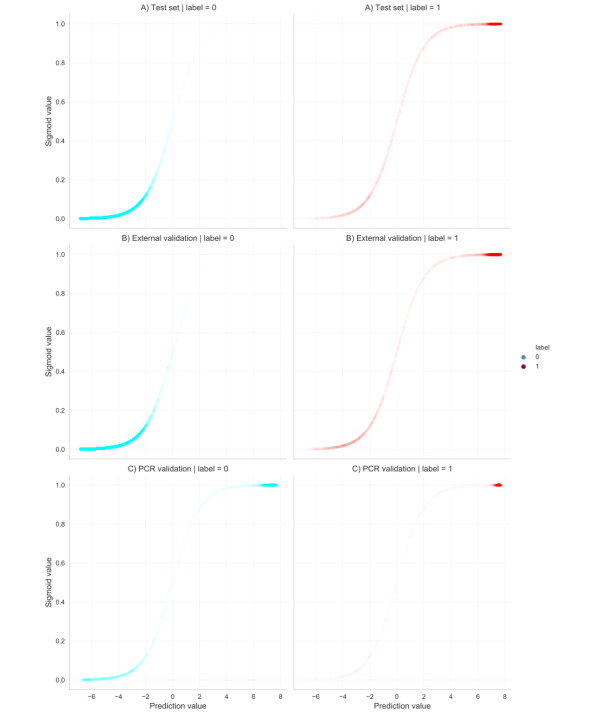
Sigmoid plots of the distribution of predictions for non–COVID-19 consultations (label 0) and COVID-19 consultations (label 1) developed as bidirectional encoder representations from transformers model on the test set (A), external validation set (B), and polymerase chain reaction validation set (C). PCR: polymerase chain reaction.

The database was partitioned into 3 sets. The training set comprised 180,215 consultations, of which 150,706 were not COVID-19–related (label 0) and 29,509 were COVID-19–related (label 1). The development and test sets each comprised 60,072 consultations: the development set included 50,279 non–COVID-19 consultations and 9793 COVID-19 consultations, while the test set included 50,377 non–COVID-19 consultations and 9695 COVID-19 consultations. The distribution of non–COVID-19 to COVID-19 consultations for all 3 sets was approximately 86:14. The model was applied to the test set after training and development. Table 1 shows the performance metrics of the developed BERT model. It achieved an overall *F*_1_-score of 0.91, precision of 0.98, and recall of 0.85, with consistent performance across both labels. Figure 2A shows the sigmoid plots for labels 0 and 1. The curve for label 0 was slightly more distinct than that for label 1, indicating that the model performed slightly better at predicting label 0.

**Table 1:** Performance metrics of the bidirectional encoder representations from transformers model on the test, external validation, and polymerase chain reaction validation sets.

	Test set	External validation	Polymerase chain reaction validation
Accuracy	0.972	0.938	0.461
Balanced accuracy	0.920	0.886	0.607
*F*_1_-score	0.907	0.870	0.352
Precision	0.98	0.990	0.223
Recall/sensitivity	0.845	0.776	0.833
Specificity	0.997	0.997	0.381

### External Validation

The model was applied to comparable data from the Rijnmond Primary Care database to assess performance on a new data set comprising 234,231 consultations: 171,603 from 2019 with ICPC codes potentially related to COVID-19 and 62,628 linked with ICPC code R83.03. Table 1 describes the performance of the BERT model on this data set. The model achieved an overall *F*_1_-score of 0.91, precision of 0.98, and recall of 0.85. Figure 2B shows the sigmoid plots for label 0 and 1. These plots were generated using a random sample of 60,000 consultations, maintaining a label 0 to label 1 ratio of 86:14. This ratio is comparable to the distribution observed in the test set, ensuring representativeness.

### Validation Using PCR Tests

The AHON database, linked with municipal health service testing data, provided the data set for PCR test validation. Out of 8987 consultations, 7408 were labeled COVID-19–negative (consultation + negative PCR test within the defined 2-week period) and 1579 were labeled COVID-19–positive (consultation + positive PCR findings within the defined 2-week period). The BERT model achieved an *F*_1_-score of 0.35, precision of 0.22, and recall of 0.83 (Table 1). Figure 2C shows the sigmoid curves for labels 0 and 1. There are relatively smooth curves and expected sigmoid shapes for label 1 predictions, suggesting that the model performed well in identifying COVID-19 consultations. However, there is an increase in the density of predictions toward the upper end of the curve for label 0, suggesting an overconfident model that makes false-positive predictions.

### Validation Before the Specific ICPC Code for COVID-19

**Figure 3:**
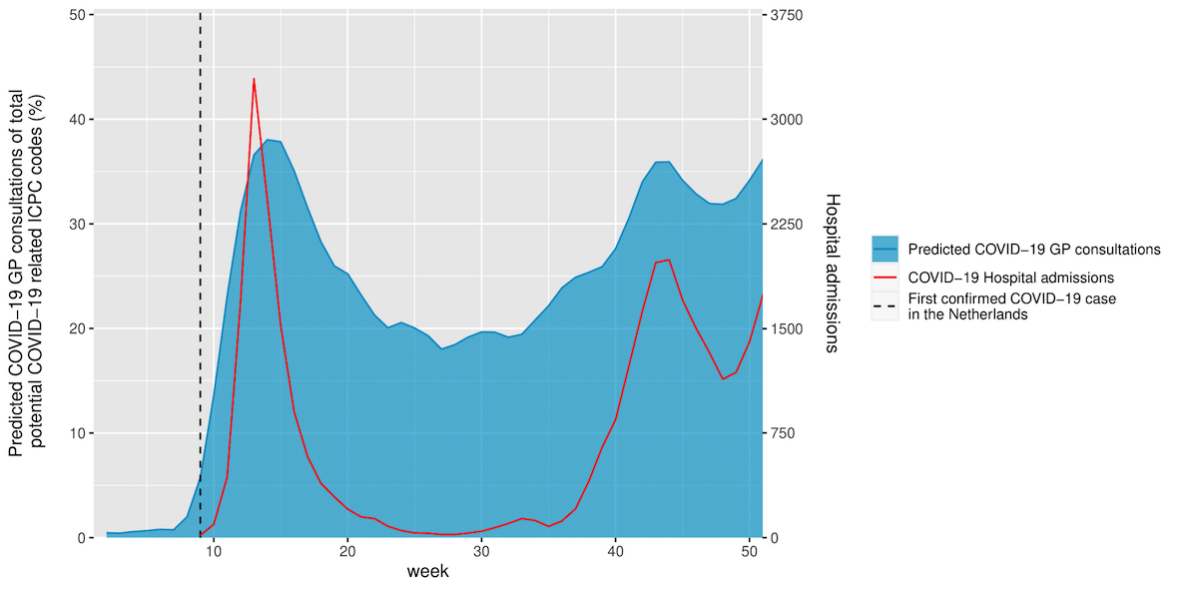
Predicted COVID-19 consultations displayed as relative to all included consultations in 2020. COVID-19–related hospital admissions are displayed in red. GP: general practitioner; ICPC: International Classification of Primary Care.

**Figure 4:**
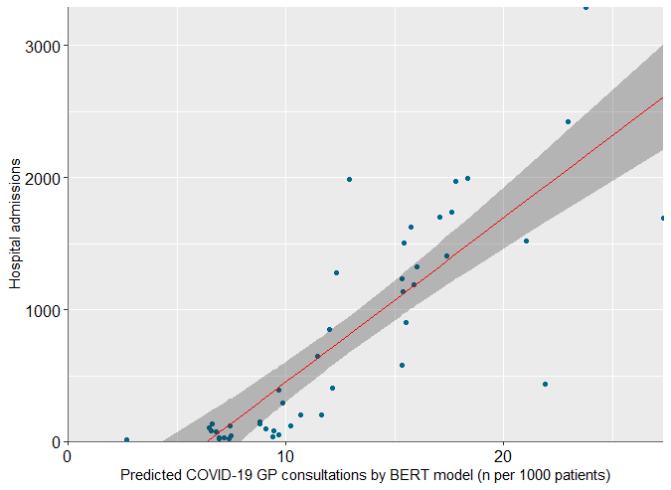
Scatterplot showing the relationship between predicted COVID-19 consultations by developed bidirectional encoder representations from transformers model and hospital admissions related to COVID-19 in the Netherlands. This plot shows the linear regression line (red) and the 95% CI (gray-shaded area). Each dot represents a weekly observation. BERT: bidirectional encoder representations from transformers; GP: general practitioner.

The BERT model’s ability to identify COVID-19 consultations during the early stages of the pandemic was evaluated by comparing its predictions for GP consultations in 2020 that had ICPC codes possibly related to COVID-19 and for national hospitalization data for COVID-19 admissions. This data set comprised 244,068 consultations. Figure 3 displays the relative number of positive predictions by the BERT model compared to the total number of consultations as well as the national hospitalization data for COVID-19 per week. This shows a higher percentage of predicted positive labels that corresponded with an increase in COVID-19 hospitalizations and a lower percentage of predictions associated with a decrease in hospitalizations. Unlike other ICPC codes potentially related to COVID-19, which showed a high frequency from January when there were no confirmed COVID-19 cases, the model’s COVID-19 predictions remained close to zero until weeks before the first confirmed case in the Netherlands (week 9) and then gradually increased over time [13]. To assess this correlation further, we performed a linear regression analysis (Figure 4). This generated a correlation coefficient (*r*) of 0.83, indicating a strong positive correlation between the variables. The reproduction script for the statistical model can be found in Multimedia Appendix 4.

The *F*_1_-score for overall significance of the model was 96.8 (1 and 43 degrees of freedom), with a *P* value of 1.411e-12, suggesting a statistically significant relationship between predicted COVID-19 consultations and COVID-19–related hospital admissions. The *R*^2^ value of 0.69 provides robust evidence of the model’s predictive power, indicating that 69% of the variance in hospital admissions can be explained by the linear relationship with the predicted cases.

## Discussion

### Main Findings

This study shows that a highly accurate BERT model can be developed to identify COVID-19–related GP consultations from EHR data in the Netherlands. The high performance metrics with the test set suggest the potential of such NLP methods to identify COVID-19 consultations accurately in general practice settings. Employing multiple validation methods further helped to describe the reliability and utility of the model in different (medical) settings.

First, external validation with a separate data set confirmed the high accuracy of the model and indicated that it is both generalizable and effective across different geographic regions with comparable EHR data. In the future, additional features could be incorporated to improve the model’s performance. This might include information about the region or the specific EHR system.

Second, the model was validated with PCR test results from before the introduction of a specific ICPC code for COVID-19, and this showed good recall but poor specificity and precision. This strengthens the findings because the model correctly identified true positives in this period. Moreover, the low precision and specificity can be attributed to the model’s inability to differentiate between negative and absent test results for COVID-19. Since the model was trained on confirmed COVID-19 cases using the ICPC code, cases with mentioned negative test results were new for the model and led to false positives.

Third, the model was applied to data from before the introduction of either nationwide testing or the specific ICPC code for COVID-19. This revealed a highly significant correlation between the model’s predicted values and true hospital admissions for COVID-19 in the Netherlands in 2020, with the number of predicted COVID-19 consultations starting to rise from week 6. Linear regression (Figure 4) confirmed that that our BERT model could significantly predict hospital admissions with a good fit, making it capable of early disease identification. The first confirmed diagnosis and hospitalization occurred in week 9 in the Netherlands [13]. Given that most patients do not require hospitalization and that hospitalization usually occurs a week after developing symptoms [15], the first patients to present with COVID-19 in general practice likely consulted their GP several weeks before the first confirmed case of COVID-19. The ability of the model to identify potential COVID-19 cases before the confirmed outbreak shows its potential for detecting infectious disease early. By analyzing the data in the weeks leading to the first confirmed case and hospitalization, the model could be developed to identify early indicators of disease and improve early detection and the response to future pandemics.

### Comparison With Previous Studies

Recent literature shows an increased use and interest in NLP for the analysis of EHRs [16]. Concerning the COVID-19 pandemic, several studies have highlighted the potential of NLP methods to detect and characterize cases from EHR data [17-20]. A large review focusing on the predictive value of COVID-19 symptoms showed that both the absence and presence of specific symptoms could accurately determine COVID-19 status [21]. NLP models can recognize unknown patterns in data, which could potentially lead to more accurate and efficient disease identification than conventional methods [4]. However, only scarce data exist regarding the development and especially the validation of NLP models in different GP care settings. This study adds to the growing body of evidence showing the potential utility of NLP methods in health care, particularly in the context of pandemic preparedness and response.

Previous research has shown that the medical use of NLP faces various barriers, including limited access to medical data, computational resources, and labeled data, together with difficulties in sharing pretrained models [22]. This study overcomes these barriers by showing the high performance of a fine-tuned, open-source, pretrained language model, with validation in comparable but different regions and time periods. Our findings suggest that the developed NLP models have the potential for broader application, which could reduce the need for additional training, computational resources, and labeling.

### Implications for Medical Settings

The results of this study suggest that automated disease identification through NLP methods can greatly improve the efficiency and accuracy of disease identification during a pandemic. These methods also have the potential to improve disease identification during regular consultations, potentially reducing treatment delays and improving outbreak prediction. The external validation has effectively demonstrated our model’s robustness and readiness for application in GP registry databases with comparable data structures. This indicates that the model has the ability to identify patients with suspected COVID-19 based on either symptoms or other findings in retrospective analyses. Nonetheless, the translation and implementation of this into GP electronic health registry systems for direct clinical feedback are yet to be explored. Further development of NLP models for disease identification could lead to its use for checking symptoms and conditions in text fields and enabling prompt action. A validated model could eventually be used to identify COVID-19 cases that may have been missed in the early stages of the pandemic when no specific tests or ICPC codes existed. This has relevance because these patients may still experience negative post-COVID outcomes, and the model could give insights into their expected disease trajectories.

The development and validation of our BERT model also highlights the potential of NLP methods to improve disease identification and management in general practice. NLP methods could facilitate more efficient and accurate diagnosis and treatment for a wide range of diseases by enabling rapid and automated analysis of large volumes of EHR data. The results of this study also support the feasibility and effectiveness of developing our BERT model to identify other diseases and conditions in general practice.

### Limitations

The developed model was trained and tested using data from general practice EHRs in the Netherlands, which may limit its generalizability and necessitate further training and validation before its application in other languages and health care systems. Using the COVID-19 ICPC codes may also have affected the model’s performance due to their limited sensitivity and specificity for identifying COVID-19 consultations. This may have resulted in missed or misclassified consultations and potential biases in the training and validation data. Additionally, differences in disease prevalence among regions or populations could affect the model’s sensitivity and specificity. Despite using multiple validation methods to address this issue, further studies are still needed to evaluate the utility of the model in real-world (medical) settings.

### Conclusions

The developed BERT model was able to accurately identify COVID-19 cases among GP consultations even preceding confirmed cases. This study demonstrates the potential of NLP methods to revolutionize disease identification in general practice, highlight their potential to identify disease outbreaks early, and improve public health outcomes. The full implications may extend beyond COVID-19 to pave the way for NLP models that can aid in the early identification of other diseases in general practice.

We showed the power of multidisciplinary efforts in harnessing technology for disease identification. Model development and validation should adopt a transparent and explainable approach that includes stakeholder involvement (including clinicians and other end users). This will not only ensure the model’s relevance and usability in real-world settings but also help ensure the development and validation of AI models in a manner that maximizes their impact on public health and outbreak management. This study therefore offers a blueprint for the early recognition of various illnesses, revealing that such models could revolutionize (infectious) disease surveillance. Continued research and innovation in the field of NLP, especially in the context of pandemic preparedness, may prove crucial for the future of health care. Our findings underscore the argument for further exploration and implementation of NLP methods to improve disease detection and management in primary care.

## Appendix

Multimedia Appendix 1 Potential acute COVID-19–related International Classification of Primary Care-1 codes.

Multimedia Appendix 2 Model card.

Multimedia Appendix 3 Characteristics of the included population from the 3 academic general practitioner networks.

Multimedia Appendix 4 Reproduction script of the statistical model.

## Abbreviations

AHON: Academisch Huisartsen Ontwikkel Netwerk
AI: artificial intelligence
BERT: bidirectional encoder representations from transformers
EHR: electronic health record
GP: general practitioner
ICPC: International Classification of Primary Care
NLP: natural language processing
PCR: polymerase chain reaction

## Data Availability

Access to the data used to train the bidirectional encoder representations from transformers model in this study can be facilitated after obtaining approval from the steering committees of the involved general practitioner networks. The data set employed in our research consists of registration texts from general practitioners, detailing sensitive and confidential information about patients’ health. However, it is important to note that the data set is not freely available; permission must be sought, along with the potential associated fees, before access is granted. These arrangements are in place to ensure proper data usage. For more information about the data set’s specifics as used in this study, inquiries can be directed to the corresponding author.
