# Supplementary material for: A Natural Language Processing Model for COVID-19 Detection Based on Dutch General Practice Electronic Health Records by Using Bidirectional Encoder Representations From Transformers: Development and Validation Study
Source: J Med Internet Res. 2023 Oct 4;25:e49944. doi: 10.2196/49944 (PMC10563863; doi:10.2196/49944)
Supplement: Multimedia Appendix 1 [file jmir_v25i1e49944_app1.pdf]

Multimedia Appendix 1: Potential Acute COVID-19 Related ICPC-1 Codes

ICPC-1 code	Description	ICPC-1 code	Description
A01	Pain general	R02	Shortness of breath/dyspnoea
A02	chills	R03	Wheezing
A03	fever	R04	Breathing problem other
A04	Weakness/tiredness general	R05	Cough
A29	General symptom/complaint, other	R07	Sneezing/nasal congestion
A77	Viral disease, other/NOS	R08	Nose symptom/complaint other
A78	Infectious disease, other/NOS	R09	Sinus symptom/complaint
B02	Lymph gland(s) enlarged/painful	R21	Throat symptom/complaint
B03	Complaints lymph gland(s)	R22	Tonsil complaints
D01	Abdominal pain/cramps general	R23	Voice symptom/complaint
D09	Nausea	R28	Limited function/disability (R)
D10	Vomiting	R29	Respiratory symptom/complaint other
D11	Diarrhoea	R74	Upper respiratory infection acute
D18	Change in faeces/bowel movements	R75	Sinusitis acute/chronic
F02	Red eye	R76	Tonsillitis acute
F70	Conjunctivitis infectious	R77	Laryngitis/tracheitis acute
H03	Tinnitus, ringing/buzzing ear	R78	Acute bronchitis/bronchiolitis
L18	Muscle pain	R80	Influenza
N01	Headache	R81	Pneumonia
N16	Disturbance of smell/taste	R83	Respiratory infection other
R01	Pain respiratory system		
