# Supplementary material for: A Natural Language Processing Model for COVID-19 Detection Based on Dutch General Practice Electronic Health Records by Using Bidirectional Encoder Representations From Transformers: Development and Validation Study
Source: J Med Internet Res. 2023 Oct 4;25:e49944. doi: 10.2196/49944 (PMC10563863; doi:10.2196/49944)
Supplement: Multimedia Appendix 2 [file jmir_v25i1e49944_app2.pdf]

Multimedia Appendix 2: Model Card

BERTje on identifying COVID-19 contacts in Dutch general practice care

- Locale: University Medical Centre Groningen
- Last Update: 26-03-2023
- Version: 1.0

Summary: A pre-trained Bidirectional Encoder Representations from Transformers (BERT) model was finetuned for binary classification of Electronic Health Records (EHR) free text fields to detect COVID-19 related contacts in Dutch general practice care.

Model type: BERT model

Parent model: BERTje: A Duch BERT model: https://arxiv.org/abs/1912.09582v1

Intended use:
- Used to identify de COVID-19 related contacts in Dutch general practice care based on EHR free text fields.

Factors:
- Input data consist of only EHR free text fields and although personal factor are not included as factor, personal factors can be described in the free text.
- The free text input was anonymized using the same tool for the training, development, test, and PCR validation sets. Text input from the external validation set was locally anonymized at the external source

Data: Data includes anonymized free text fields from EHR from the year 2019 with at least one of 49 suspicious COVID-19 ICPC codes and EHR with ICPC code R83.03 (COVID-19) from the years 2020 and 2021. Exclusively Lab results and ICPC texts are removed from the free text fields.
Records originated from 2019 were labeled COVID-19 negative, while records from 2020 onwards were labeled as COVID-19 positive. The dataset was divided into a training set (60%), model selection set (20%), test set(20%).

Table S1: *Distribution of Covid-19 labels across used datasets.*

Dataset	Covid-19 positive (n%)	Covid-19 negative (n%)	Total
**Training set**	29.509 (15)	150.706 (85)	180.215
**Development set**	50.279(15)	9.793(85)	60.072
**Test set**	50.377(15)	9.695 (85)	60.072
**External validation**	62.628 (27)	171.603(73)	234.231
**Validation using PCR-tests**	7.408 (82)	1.579 (18)	8.987

Training: During the training development of the model, various parameters were tested, including positive weight, batch size, and learning rate. A total of 350 consultations were validated by two medical doctors (MDs). Based on the results, no further adjustments were deemed necessary.

Results:

***Table S2:** performance metrics of the BERT-model on the test set, the external validation set and the validation set using PCR-tests.*

	Test set	External validation	Validation using PCR-tests
**Accuracy**	0,972	0,938	0,461
**Balanced accuracy**	0,920	0,886	0,607
**F-1 score**	0,907	0,870	0,352
**Precision**	0,98	0,990	0,223
**Recall/ sensitivity**	0,845	0,776	0,833
**Specificity**	0,997	0,997	0,381

Ethical considerations:

Data: The model utilizes electronic health record (EHR) data, which has been anonymized. The research was assessed by the medical ethics committee as non WMO, indicating compliance with ethical guidelines and regulations.

Appropriate use: The model is designed to identify COVID-19 related contacts using registry data. Currently, it is not intended as a standalone tool for clinicians to identify patients with COVID-19 before the introduction of the COVID-19 specific ICPC code or testing.

Risks: Potential risks and harms, such as misdiagnosis or overreliance on the model, are acknowledged. To mitigate these risks, appropriate human oversight is essential to ensure proper interpretation and decision-making based on the model's outputs.

Inappropriate Settings: The model should not be used in settings where the data or context significantly deviates from the training distribution, as this may compromise its performance and reliability.

Generalizability: The model's generalizability is primarily applicable to similar general practice settings with comparable electronic health record systems and data structures. However, it is important to note that the model's performance may vary when applied to datasets from different geographic regions or healthcare contexts. Handling data outside its training distribution poses limitations, and further evaluations and adaptations may be necessary for successful deployment in new settings.

Caveats and Recommendations:

Further Testing: The results of this study may suggest the need for additional testing and evaluation to improve the model's performance. Specifically, exploring methods to enhance the model's ability to differentiate between cases with negative COVID-19 test results and those without any test results would be beneficial.

Model Use Recommendations: It is recommended to use the model as a supportive tool for researchers rather than as a substitute for clinical judgment. Incorporating appropriate human oversight and expertise is crucial for responsible and effective utilization of the model.

Contact information:

- Maarten Homburg, t.m.homburg@umcg.nl
- Eline Meijer, e.n.meijer@umcg.nl
