# Supplementary material for: A Natural Language Processing Model for COVID-19 Detection Based on Dutch General Practice Electronic Health Records by Using Bidirectional Encoder Representations From Transformers: Development and Validation Study
Source: J Med Internet Res. 2023 Oct 4;25:e49944. doi: 10.2196/49944 (PMC10563863; doi:10.2196/49944)
Supplement: Multimedia Appendix 3 [file jmir_v25i1e49944_app3.pdf]

Multimedia Appendix 3: Characteristics of Included Population From the Three Academic GP Networks

	UMCG<br>N (%)	RUMC<br>N (%)	MUMC<br>N (%)
**Population**	184 700 (100)	31 351 (100)	87 499 (100)
**Gender**			
MALE	78 658 (43)	13 127 (42)	36 715 (42)
FEMALE	106 042 (57)	18 224 (58)	50 784 (58)
**Age (years)**			
0-17	35 847 (19)	8 574 (27)	15 661 (18)
18–40	44 495 (24)	9 143 (29)	20 503 (23)
40–64	60 073 (33)	9 578 (31)	29 789 (34)
>65	44 285 (24)	4 056 (13)	21 546 (25)

Abbreviations: GP, general practitioner; MUMC, Maastricht University Medical Center; RUMC, Radboud University Medical Center Nijmegen; UMCG, University Medical Center Groningen
