# Supplementary material for: A Natural Language Processing Model for COVID-19 Detection Based on Dutch General Practice Electronic Health Records by Using Bidirectional Encoder Representations From Transformers: Development and Validation Study
Source: J Med Internet Res. 2023 Oct 4;25:e49944. doi: 10.2196/49944 (PMC10563863; doi:10.2196/49944)
Supplement: Multimedia Appendix 4 [file jmir_v25i1e49944_app4.pdf]

Multimedia Appendix 4: Reproduction Script of Statistical Model

```
#libraries
library(tidyverse)

## Warning in as.POSIXlt.POSIXct(Sys.time()): unable to identify cur
rent timezone 'C':
## please set environment variable 'TZ'

## -- Attaching packages --------------------------------------- tid
yverse 1.3.2 --
## v ggplot2 3.3.5     v purrr   0.3.4
## v tibble  3.1.8     v dplyr   1.0.8
## v tidyr   1.2.0     v stringr 1.4.0
## v readr   2.1.2     v forcats 1.0.0
## -- Conflicts ------------------------------------------ tidyverse
_conflicts() --
## x dplyr::filter() masks stats::filter()
## x dplyr::lag()    masks stats::lag()

#load data
covid_hospital_admissions <- readRDS("Z:/3_Projects/AI-model/1_data/
Ziekenhuisopnames/COVID_19_ziekenhuisopnames_per_week.rds")

predicted_covid_consults_per_week <-  readRDS("Z:/3_Projects/AI-mode
l/1_data/predicted_cov_consultation_per_week.rds")
```

<u>Create Linear model Hospital admissions vs. COVID-19 consultations:</u>

```
cor_ziekhuis <- covid_hospital_admissions %>% filter(year == 2020)

cor_model <- predicted_covid_consults_per_week %>%
  filter(year == 2020 & week_iso > 8 & bin_pred == "1")

cor_df <- cor_model %>% left_join(cor_ziekhuis)

## Joining, by = c("week_iso", "year", "Hospital_admission")

cor(cor_df$n_covid_per_1000, cor_df$Hospital_admission)

## [1] 0.8321139

linear_model <- lm(Hospital_admission ~ n_covid_per_1000, data = cor
_df)
summary(linear_model)

##
## Call:
## lm(formula = Hospital_admission ~ n_covid_per_1000, data = cor_df
)
```

```
##
## Residuals:
##      Min       1Q   Median       3Q      Max
## -1499.50  -240.86    -9.17   145.82  1170.12
##
## Coefficients:
##                  Estimate Std. Error t value Pr(>|t|)
## (Intercept)       -794.27     174.42  -4.554 4.30e-05 ***
## n_covid_per_1000   124.41      12.65   9.839 1.41e-12 ***
## ---
## Signif. codes:  0 '***' 0.001 '**' 0.01 '*' 0.05 '.' 0.1 ' ' 1
##
## Residual standard error: 459.5 on 43 degrees of freedom
## Multiple R-squared:  0.6924, Adjusted R-squared:  0.6853
## F-statistic:  96.8 on 1 and 43 DF,  p-value: 1.411e-12
```

Plot Linear model Hospital admissions vs. COVID-19 consultations:

```
cor_df$fitted <- linear_model$fitted.values

temp <-  data.frame(predict(linear_model, cor_df, interval = "confid
ence"))
cor_df$ci_lwr <- temp$lwr
cor_df$ci_upr <- temp$upr

g <- ggplot(cor_df, aes(x = n_covid_per_1000, y  = Hospital_admissio
n)) +

  geom_point(color = "deepskyblue4") +
  geom_ribbon(mapping = aes(x= n_covid_per_1000,
                            ymax = ci_upr,
                            ymin = ci_lwr),
              alpha = 0.3) +

  scale_y_continuous(expand = c(0,0)) +
  scale_x_continuous(expand = c(0,0)) +

  geom_line(aes(y = fitted), color = "red") +

  labs(x = "Predicted COVID-19 GP consultations by BERT model (n per
1000 patients)",
       y = "Hospital admissions") +
  theme(axis.text =  element_text(size = 12, color = "black"),
        axis.line = element_line(size = 0.5, color = "grey40")) +
  coord_cartesian(xlim = c(0, NA), ylim = c(0,NA))

g
```
